# Comment on Frye et al. Air Pollution and Maximum Temperature Are Associated with Neurodevelopmental Regressive Events in Autism Spectrum Disorder. *J. Pers. Med.* 2022, *12*, 1809

**DOI:** 10.3390/jpm15080381

**Published:** 2025-08-15

**Authors:** Keith Fluegge, Kyle Fluegge

**Affiliations:** Institute of Health and Environmental Research, Columbus, OH 43235, USA; kyle.fluegge@gmail.com

Frye et al. [1] have presented evidence that air pollution may spur neurodevelopmental regression (NDR) among those diagnosed with autism spectrum disorders (ASD). NDR is an event, which may manifest as fever of unknown origin (FUO) and seizures, in which a child loses previously acquired skills and displays an autistic phenotype with or without a specific triggering event. The authors linked exposure to air pollution, and specifically PM2.5, and temperature to NDR with and without a triggering cause. We appreciate Dr. Frye’s time and his subsequent letter, further clarifying the complexity of the role of air pollution in ASD risk.

Our prior work, using two-way fixed effects regression approach, has linked the farm use of synthetic nitrogen (as the most recognized causal contributor to environmental nitrous oxide [N_2_O] burden) to a myriad of disorders often comorbid to ASD, including ADHD [2], epilepsy [3], inflammatory bowel diseases [4], drug dependence [5], gut microbiome alterations [6], and dementia [7]. While N_2_O is well known as an analgesic in medicine/dentistry, the compound has a critical duality that has largely been ignored by the medical establishment. N_2_O is a greenhouse gas and an air pollutant that is principally derived from synthetic nitrogen application in agricultural management but also mobile and stationary combustion sources and bodies of water.

IHER’s prior analyses also controlled for common air pollutants measured by the E.P.A. Air Quality System, as Frye et al. [1] have done, yet we were unable to document any significant associations with these variables. Only farm use of synthetic nitrogen fertilizers, a variable not captured by E.P.A. surveillance, was the most significant factor, increasing contemporaneous hospitalization risk for ADHD (i.e., representing the most severely impaired phenotype) and lagged mortality risk for Alzheimer’s disease, while decreasing contemporaneous hospitalization risk for epilepsy and inflammatory bowel disease. These findings are strongly consistent with the known cognitive and neurological effects from N_2_O exposure, even at trace levels, leading us to conclude that environmental emissions of N_2_O were a primary cause of neurodevelopmental disorders and their highly comorbid manifestations.

The present authors have addressed the previous findings of Frye et al. that BH4 (tetrahydrobiopterin) treatment in ASD may improve metabolic outcomes and simultaneously restore myogenic and central catecholaminergic activity, like the effect of an opioid antagonist [8]. Such a therapeutic action may, therefore, indicate an opiate-dependent state in ASD induced from air pollution exposures, again implicating N_2_O emissions as an etiological factor.

N_2_O is believed to target the opioidergic system through its induction of dynorphin (DYN) release and activation of the kappa opioid receptor (KOR), an opioid receptor subtype implicated in negative stress adaptation and impulsivity. Behavioral stereotypies are dysregulated under conditions of trace N_2_O treatment [9], presumably due to N_2_O-induced activation of the DYN/KOR system. Additionally, N_2_O-induced differences in growth patterns [10], altered body laterality [11], reduced peak velocity of voluntary eye saccades [12], bowel distension [13], and greater middle ear pressure (which can reduce sound sensitivity) [14] may also reflect heightened opioidergic activity, and mirror, almost exactly, the unique morphological and physiological manifestations found in ASD. Interestingly, N_2_O has also been linked to both clinical outcomes of NDR: that is, FUO and seizure.

Behavior-mediated hypothermia was the initial response to N_2_O inhalation in rats. However, chronic exposure resulted in thermogenesis. That is, chronic exposure to 60% N_2_O elicited a compensatory hyperthermic state as indicated by core temperature, which was mediated by an increase in endogenous heat production, described by the authors as a drug-induced allostasis [15]. Additionally, clinical evidence supports the role of N_2_O as an agent of thermogenic control, showing postoperative fever is significantly associated with other complications resulting from the use of N_2_O in general anesthesia [16]. Both clinical case reports and animal studies have also documented the N_2_O-induced suppression of epileptiform activity, while tolerance/withdrawal to N_2_O induces seizures [17,18].

Our characterization of ASD as a disorder of opioid dysregulation is a view previously shared by others [19] and further supports our theory of environmental N_2_O as a primary cause of ASD. Indeed, a comprehensive autistic phenotype was induced in mu opioid receptor null mice, suggesting the critical importance of KOR in characterizing ASD syndrome [20]. We are not aware of any report documenting such a profound influence of particulate pollution on the opioidergic system.

We have also highlighted climatological influence on ASD risk. Both moderate rainfall events and hurricanes have been linked to ASD risk [21], reflecting the finding that precipitation (irrespective of intensity) robustly increases N_2_O emissions, though nitrate supply and soil carbon can be limiting factors to emissions [22]. The significant interaction between duplication burden and ozone exposure in ASD risk may be confounded by the role of N_2_O in both ozone stasis [23] and plant breeding due to enhanced chromosomal duplication [24]. The converged models from Frye et al. [1] that temperature and PM2.5 are linked to NDR also support the theory that environmental N_2_O burden drives ASD risk, since synthetic nitrogen fertilization programs increase not only N_2_O emissions but also PM2.5 emissions, and temperature significantly affects N_2_O emissions [25,26]. The contextual significance of these associations may be that enhanced PM2.5 emissions and reduced temperature reflect decreasing N_2_O flux and thus exposure among ASD subjects, which may reflect an opioid withdrawal and/or tolerance and trigger NDR events, including but not limited to FUO (otherwise known as drug-induced allostasis) and seizure. Therefore, we suggest that while these pollution variables may often share a common source, each may exert a unique biological impact.

Furthermore, the fact that Frye et al. continue to publish therapeutic interventions in ASD that rescue N_2_O deficits is remarkable [27,28]. The authors have published in this journal the effectiveness of both leucovorin (folinic acid) and B12 in the treatment of ASD symptoms. N_2_O dysregulates both folate and cobalamin metabolism. Briefly, in the folate-mediated one carbon metabolic pathway, a carbon unit from serine or glycine is transported to tetrahydrofolate (THF), which yields methylene-THF, a key endogenous one-carbon donor for the synthesis of thymidine, which is incorporated into DNA. Methylene-THF can also be reduced to 5-methyl-THF by methylenetetrahydrofolate reductase (MTHFR gene) which can be used to methylate homocysteine to form methionine. The latter reaction is catalyzed by a B12-containing methionine synthase. However, N_2_O exposure irreversibly oxidizes the cobalt moiety within B12 and thereby inactivates MS enzyme and induces cobalamin and folate insufficiencies, especially under conditions of chronic and/or prolonged exposure. It is this metabolic insult that could explain why cobalamin and folate supplementation improve metabolic and clinical outcomes in ASD, as Frye et al. have now reported. Interestingly, leucovorin has been shown to rescue bone marrow depression among N_2_O-exposed subjects, presumably through restoration of folate metabolism [29].

**Figure 1:**
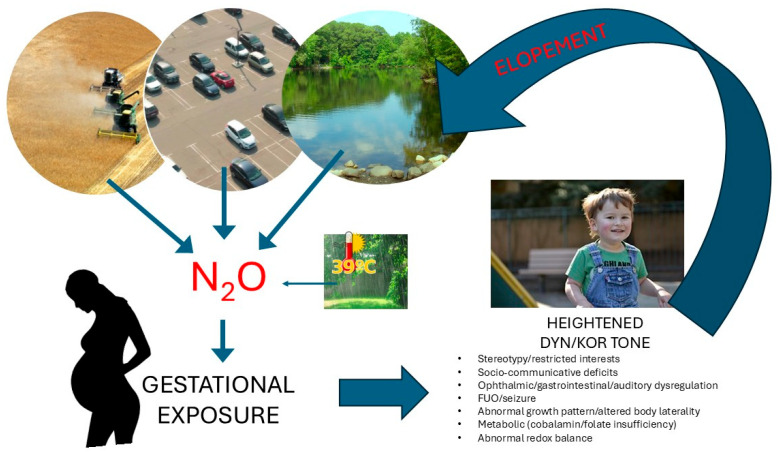
The figure depicts IHER’s hypothesis that gestational exposure to environmental emissions of N_2_O from varying sources, including farm use of synthetic nitrogen fertilizers, mobile and stationary combustion sources, and bodies of water, increases risk of ASD. Temperature and precipitation also serve as ancillary weather-related patterns that may enhance N_2_O emissions from heavily synthetically fertilized soils. We hypothesize that brain remodeling during gestational exposure to environmental N_2_O creates an elevated DYN/KOR system in ASD brain. N_2_O induces acute elevations in striatal dopamine output, and this is countered by a heightened DYN/KOR tone. It is this specific opioid subtype that results in the hallmark clinical deficits among ASD subjects, including symptoms of behavioral stereotypy, restricted interests, socio-communicative deficits, ophthalmic, gastrointestinal, auditory, and sensory dysregulation. The DYN/KOR system has also been implicated in reduction in epileptiform activity and thermogenic control, indicating that FUO and seizure could represent a tolerance and/or withdrawal from N_2_O among ASD subjects, otherwise pathologized as NDR. We would suggest that NDR simply reflects a brain highly developed to withstand chronic environmental N_2_O emissions and that N_2_O exposure would reverse NDR, perhaps explaining the phenomenon of elopement, as depicted in the figure. Elopement behavior is an increasingly documented phenomenon in ASD. Kiely et al. [30] have characterized data from the CDC “Pathways” Survey analysis by showing that wanderers scored higher on five of six subscales of the Children’s Social Behavior Questionnaire. Other characteristics of wanderers included shifts in mood, not recognizing dangerous situations, easily panicked, and quick to anger and over-reaction. These characteristics of elopement may lead to situations wherein ASD children experience an increased risk for bodily harm and injury. Anderson et al. [31] studied the impact of elopement on families of children with autism, finding children with ASD who went missing for long periods of time had increased risk for drowning and traffic injury. It is not clear why children with ASD engage in elopement behavior. However, both situations place ASD subjects at concentrated sources of environmental N_2_O emissions (i.e., bodies of water and combustion sources). The question as to whether NDR, including the inclination to elope, in ASD represents a state of drug-induced allostasis needs to be explored further.

Given the evidence thus far, we would reiterate to the authors that they acknowledge the potential confounding of their findings by exposure to trace levels of the air pollutant, N_2_O, which likely induces a kappa opioid-dependent state in ASD that may mediate many of the socio-communicative/behavioral deficits, metabolic, ophthalmic, gastrointestinal, auditory dysregulation seen in the disorder. NDR may reflect a N_2_O withdrawal and/or tolerance state in ASD brain (characterized by epileptiform activity, FUO, etc.), in which a highly developed kappa opioidergic tone is developed during gestational exposure to N_2_O and requires satiation, be it through environmental or clinical means. Whether administration of therapeutic N_2_O or permitting ASD subjects to wander to sources of environmental N_2_O pollution (i.e., elopement to bodies of water, Figure 1) would ameliorate NDR is an ethically dubious inquiry that warrants clarification, especially given the clinical push to begin therapeutic N_2_O for various affective disorders. The transient, antidepressant effects of N_2_O require chronic treatment, which poses a risk to folate and cobalamin metabolism over time, as described.

To further explore our theory that ASD represents an opioid dependence, we test whether statewide use of nitrogen fertilizers, both farm and nonfarm [32], is associated with hospitalizations for ASD from 1997 to 2006 recorded in the Healthcare Cost and Utilization Project Net [33] and seeking to control relevant contemporaneous covariates implicated in ASD including maternal obesity [34] and precipitation [35]. Consistent with our prior methodologies [2,3,4,5,6,7], we only include identified covariates if a statistically significant relationship (*p* < 0.05) was independently found using ordinary least squares regression in any year of the panel data (1997–2006). Briefly, a random variable Y is said to have a Poisson distribution with parameter *μ* if it takes integer values *y* = 0, 1, 2, … with probability

$$Pr\left\{Y=y\right\}=\frac{e^{-\mu }\mu^{y}}{y!}$$

for μ > 0. The mean and variance of this distribution can be shown to be E(Y) = var(Y) = μ. We have a sample of *n* observations of discharges related to ASD, *y*_1_, *y*_2_, …, *y_n_,* which are treated as realizations of independent Poisson random variables, with *Y_ij_*, *~P*(*μ_ij_*), where *i* represents a state and *j* an observation year. We let the logarithm of the mean depend on a vector of time-varying explanatory variables (i.e., farm and nonfarm use of nitrogen fertilizers), *x_ij,_* such that the log-linear model is the following: log (*μ_ij_*) = *x_ij_′β_1_*. Exponentiating, we have a multiplicative model for the mean discharges: *μ_ij_* = exp{*x_ij_′β_1_*}. In each case, the exponentiated regression coefficient exp{*β_1ijk_*} yields an incidence rate ratio (IRR), which represents a multiplicative effect of the *k*th predictor on the mean. Increasing *x_k_* by one-log unit multiplies the mean by a factor exp{*β_1k_*}. Prior reports using this methodology indicated that farm use of synthetic nitrogen fertilizers increased risk of hospitalization with an ADHD diagnosis and decreased hospitalization risk for epilepsy and inflammatory bowel disease. Because we believe ASD is derived from gestational exposure to environmental N_2_O emissions and results in brain remodeling to withstand this level of lifetime exposure, we suspect that farm use of synthetic nitrogen will reduce hospitalization with an ASD diagnosis.

We have also systematically searched news reports to identify cases of wandering and missing ASD children. We searched Google News using the terms “autism,” “children,” “lost,” from the period 1 June 2015 to 5 October 2016. All retrieved news reports were reviewed for information regarding the last place the ASD subject was seen and the location where the ASD subject was found. News reports outside of the United States were excluded, as were reports where elopement was not the outcome of interest or it was not clear elopement had occurred or occurred outside the time period of interest. Reports were screened for duplications, which were excluded. A limitation of this search is the uncertainty of an ASD diagnosis among the subjects. However, many news reports interviewed family members who publicly indicated the ASD diagnosis of the subject in question.

The results of our present analyses support prior exploratory investigations associating use of the herbicide, glyphosate, and nitrogen fertilizers with adverse mental health outcomes and specifically an increase in all-listed ADHD hospital discharges. Herein, we report that the contemporaneous statewide farm use of nitrogen fertilizers significantly protects against hospitalization for autistic disorder (*p* < 0.001) (Table 1). Identified covariates were not added in the regression models since they did not meet our threshold requirement for inclusion. The current finding contrasts with the association between farm use of nitrogen fertilizer and increasing risk of hospitalization for ADHD [2], suggesting that ASD may reflect an opioid-dependent state developed during chronic, gestational exposure to environmental N_2_O emissions. Our systematic, qualitative review of news reports identified 43 cases of possible elopement among ASD subjects. We excluded eight news items (N = 9) since they did not meet our specified criteria (N = 4, not located in the United States; N = 3, not clear elopement had occurred; N = 1, duplicate report; N = 1, report of a case outside the timeline). Most news reports characterized elopement in ASD as wandering off to outside locations, frequently areas of vehicular traffic and near bodies of water (Table 2).

**Table 1:** Incident rate ratio of hospitalization for autistic disorder associated with a one log-unit change in statewide farm and nonfarm use of nitrogen fertilizers using state and time fixed effects Poisson regression in R (packages ggplot and sandwich with robust standard errors) for all available HCUPnet states, United States, 1997–2006. Two-way linear fixed effects is a well-known method for estimating causal effects from panel data. Regression diagnostics checked using visual inspection of residual plots.

Pollutant (State-Time bs)	Farm		Nonfarm	
	IRR	95% CI	IRR	95% CI
ASD (ICD9:299.0)	0.91	0.85–0.98 *	1.04	0.99–1.08

* *p* < 0.025, adjusted for multiple pollutant comparisons (0.05/2 = 0.025).

**Table 2:** The de-identified results of a systematic search of public news reports identifying cases of elopement in autism. Details gathered from the reports include the location the ASD subject was last seen, if known, and the location the subject was found.

Case	Last Seen	Found
1	Cornfield	Marshy area
2	--	Expressway
3	--	Drowned
4	--	Creek area (clothing)
5	Classroom	Expressway
6	Pharmacy	Expressway
7	Hotel	Drowned
8	Home	Drowned
9	Home	Expressway
10	Home	Grassy ravine
11	Home	--
12	Home	Traffic intersection
13	--	Railroad Station
14	School	Restaurant
15	Recreation center	Drowned
16	Home	Traveling (Bus)
17	Home	Parkway
18	Home	Drowned (pool)
19	Clinic	Residential yard
20	Home	Hit by train (Death)
21	Train	--
22	--	Drowned (pool)
23	Home	Neighbor’s home
24	--	Park
25	Restaurant	Drowned
26	--	Busy street
27	Bus	Busy street
28	--	Heat-related death
29	--	Busy street
30	--	Drowned (pool)
31	--	By a motorist
32	School	Busy Street
33	--	Busy street
34	Home	Farm

A brief word of caution about these findings is warranted, though, since the use of hospitalizations is not an accurate prevalence rate. There is a potential for misclassification bias in our dependent variables of interest. We may be capturing the most severe cases of neurodevelopmental disability. Though news reports indicating the propensity of ASD subjects to elope to outside locations recognized as acute hotspots of environmental N_2_O further facilitates the hypothesis that elopement in ASD and related disorders may be a drug-seeking activity. Future work ought to better study why elopement in ASD occurs and its relation to NDR, specifically.

In the time since our first commentary on Dr. Frye’s work in 2016, the U.S. Environmental Protection Agency has documented “considerable” indirect N_2_O emissions from wastewater drainage systems in Ohio city streets [36]. Our Institute has begun preliminary measures of direct emissions from combustion sources, which are expectedly higher. Cumulatively, these exposures could reach trace levels that have been shown to induce cognitive deficits in human subjects [37] and significant changes in neurotransmission in animal models of N_2_O-induced neurotoxicity [38]. It is, therefore, not appropriate for Dr. Frye to cite E.P.A. data limitations in his defense: the data are quite alarmingly revealing, albeit when studied. The bigger question is why these findings have not been expanded upon and translated into the Air Quality System. Why has Dr. Frye, as a leading national expert in autism research, ignored such findings and not pushed for such action, given his published reply to our novel hypothesis regarding N_2_O and its dynorphinergic-mediated inhibition of alpha 7 nicotinic acetylcholine receptor and nitric oxide synthase (NOS) uncoupling [8,39]?

Dr. Frye seems keen to build a research portfolio based on the theory that N_2_O induces ASD by pursuing regulatory approval for multiple candidate therapeutics that explicitly rescue N_2_O deficits like neuronal NOS modulators [40], cobalamin, and leucovorin. Yet, he seems unwilling to acknowledge our theoretical framework that has informed this work since 2016. Respectfully, the complexity that Dr. Frye mentions seems to reflect more of a bureaucratic inefficiency on the part of the E.P.A. and a focus on building his clinical practice and publication record rather than any real difficulty in parsing simple biological realities. The broader incentive structure in chronic disease research, including ASD, remains concerning.

Therefore, the purpose of this commentary is to let the public at large, and most especially parents of children with ASD, know what Dr. Frye seems to only want to tacitly acknowledge. That is, environmental emissions of N_2_O, derived primarily from synthetically driven agricultural systems and mobile/stationary combustion sources, among many other sources, plausibly induce the hallmark clinical symptoms, including NDR, and metabolic aberrations seen in the disorder. The N_2_O pollution theory of causation also explains many of the other environmental factors that have been implicated in ASD, including rainfall/hurricanes, ozone, particulate matter pollution, etc.

Our Institute’s preliminary direct N_2_O emissions testing supports earlier scientific findings from the E.P.A. that such emissions are quantifiable and are ubiquitously present at levels known to impair cognitive functioning in human subjects and alter neurotransmission in animal models of N_2_O neurotoxicity. To build upon our prior published epidemiological associations [2,3,4,5,6,7,8], our Institute has begun a program of environmental assessments of individual exposure to N_2_O. We have advised for many years that comprehensive environmental monitoring of N_2_O emissions should be conducted and that remains our recommendation, even more so today, given the data that have emerged since our initial introduction of this hypothesis [2,3,4,5,6,7,8].
